# Performance evolution of prestressing anchor bars in corrosive environments experimental study

**DOI:** 10.1038/s41598-023-35577-8

**Published:** 2023-06-02

**Authors:** Dandan Liu, An Chen

**Affiliations:** 1 Faculty of Land Resources Engineering, Kunming University of Science and Technology, Kunming, 650093 China; 2 School of Geography and Land Engineering, Yuxi Normal University, Yuxi, 653100 China; 3 Key Laboratory of Geohazard Forecast and Geoecological Restoration in Platea Mountainous Area, Ministry of Natural Resources of the People’s Republic of China, Kunming, 650093 China

**Keywords:** Environmental sciences, Engineering, Materials science

## Abstract

In order to explore the performance evolution of prestressed anchor cables in corrosive environment, the corrosion immersion and corrosion damage performance tests of prestressed anchor bars in corrosive environment were carried out indoors. Based on the experimental results, the effects of stress level, pH and time on the corrosion process of prestressing anchor bars, the amount of corrosion per unit length and the change of mechanical properties were analyzed. It was found that the greater the stress level in the three corrosive media, the more severe the corrosion of the anchor bars, especially in acidic solutions; at the beginning of corrosion, prestressing anchor bars corrode fastest in neutral solution, followed by acidic and slowest in alkaline, with the continuation of corrosion, the degree of corrosion damage to anchor bars by acidic solution is more obvious; The corrosion damage of anchor tendons reflected by the corrosion amount per unit length is uniform corrosion, and local corrosion will aggravate the degree of corrosion damage of prestressing anchor tendons; The longer the corrosion time, the more serious the corrosion of anchor bars, and the 2 mechanical indexes of anchor bar elongation and fracture load have a significant decreasing trend.

## Introduction

Prestressed anchorage technology has been widely used in various geotechnical projects due to many advantages such as improving the stability performance of the geotechnical body itself, reducing the volume of the structure and reducing the self-weight of the structure, effectively controlling the deformation of geotechnical engineering and lower engineering cost^1–5^. However, in the actual project, the prestressing anchorage engineering structure is located in a very harsh environment, and the prestressing anchor will be subject to electrochemical corrosion and stress corrosion by the corrosion medium in the groundwater and geotechnical body, and the corrosion damage makes the stability and structural strength of the anchorage engineering structure seriously reduced or failed^6,7^. At present, there have been many cases of corrosion damage in China, such as the stress corrosion damage of some steel strands found after 6–8 years of use in three prestressing anchor cables in Meishan Reservoir, Anhui, China^8–11^; two slopes in the southern section of the Beijing-Zhuhai Expressway in Guangdong were destabilized by slope failure due to corrosion of prestressing anchor cables 2 years after opening to traffic^12^; the collapse of the Rainbow Bridge in Qijiang County, Sichuan, which was also caused by corrosion damage^13–15^. The damage caused by corrosion of prestressing anchorage engineering structures is more hazardous and should not be ignored.

Wu et al. developed an anchor cable stress corrosion cracking simulation test device and explored the subcritical crack expansion characteristics after stress corrosion of anchor cables using scanning electron microscopy, and pointed out that the stress level was negatively correlated with the anchor cable failure time^16^. Gamboa and Atrens studied the critical stress corrosion values and stress corrosion mechanisms of different steels anchor bolts based on the prestressed anchor bolts that have been in service for many years in a coal mine in Australia^17^. Wang et al. studied the stress, corrosive environment, and time effects on the stress corrosion of grouted rock anchors, and pointed out that the NaCl solution was more corrosive than the Na_2_SO_4_ solution, time had the most important effect on the corrosion effect of anchor bars, and the corrosive environment had relatively little effect on the bond strength of grouted rock anchors^18^. Zhang et al. investigated the corrosion damage morphology and microscopic corrosion damage evolution mechanism of prestressed anchor tendons based on indoor corrosion tests using ultra-deep field 3D digital microscopy and scanning electron microscopy^19^. Wang et al. analyzed the time-varying behavior of corrosion damage of prestressing anchor tendons in weak acid and oxygen flux conditions and the relationship between anchorage force and corrosion rate of prestressing anchor tendons in this environment by conducting indoor accelerated corrosion tests with the aid of an electrochemical test system^20,21^. Zhu et al. analyzed the relationship between factors such as prestress, pH and environmental oxygen concentration and the corrosion rate, appearance damage characteristics, loss per unit length, and mechanical properties of anchor tendons through indoor prestressing anchor tendon immersion corrosion tests and corrosion specimen material mechanical properties tests^22^. Zheng et al. used anchor ropes with comparable stress levels and similar corrosive environments to the actual site to study the corrosion rate of anchor bars, the variation law of mechanical properties with time, and the degree of influence of stress corrosion^23^.

Prestressed anchorage engineering structures are and will be subjected to stress corrosion for a long time, and relatively few studies have simulated the evolution of prestressed anchor performance in complex geotechnical environments^24,25^. Therefore, it is of great theoretical significance to explore the corrosion law and performance damage law of prestressed anchorage structure under the joint action of corrosion factors and mechanics by suitable accelerated corrosion experimental methods.

In this paper, the immersion test of prestressing anchor tendons in corrosive environment and the mechanical properties test of corrosive anchor tendons were carried out andbased on the experimental results, the relationship between the stress level, pH value and time and the corrosion process of anchor bars, the corrosion amount per unit length and the change of mechanical properties was analyzed. The obtained performance evolution laws of prestressing anchor bars can provide some reference basis for the evaluation of the service life and safety of prestressing anchorage structures under actual working conditions.

## Evolutionary test of prestressing anchor performance

### Corrosive environment design

To simulate different corrosion conditions, three main factors, namely stress level, pH and time, were set up to carry out indoor corrosion tests on anchor bars under different corrosive environments. In this test, 24% (100 MPa), 36% (200 MPa) and 48% (300 MPa) of the design ultimate tensile strength were used as the anchor bar test load, while no prestressing anchor bars were set for comparison. The pH was set at three levels of 5, 7 and 9 to study the development of corrosion of anchor tendons at pH values ranging from acidic to alkaline; the acid solution is diluted with concentrated hydrochloric acid and tap water, and the alkaline solution is diluted with sodium hydroxide and tap water. A total of two time periods were chosen for corrosion, 3 and 6 months, respectively. The corrosion factor level table is shown in Table 1.

**Table 1:** Corrosion test factor level table.

Level	Corrosion factors		
	Stress level	pH value	Time (months)
1	0	5	3
2	24% (100 MPa)	7	6
3	36% (150 MPa)	9	
4	48% (200 MPa)		

According to the purpose of the experimental research on corrosion factors for the combination of design, specific test plan Table 2, the test a total of 72 anchor bar specimens, of which 54 for the application of prestressed anchor bars, 18 for the non-prestressed anchor bars. The number in the table represents the test chamber number where the anchor bar specimen is located.

**Table 2:** Corrosion test plan.

Test chamber number	Stress level	pH value	Time (months)
1.4.7	0 24% (100 MPa) 36% (150 MPa) 48% (200 MPa)	5	3
2.5.8		7	
3.6.9		9	
10.13.16	0 24% (100 MPa) 36% (150 MPa) 48% (200 MPa)	5	6
11.14.17		7	
12.15.18		9	

### Corrosion test device and test system

**Figure 1:**
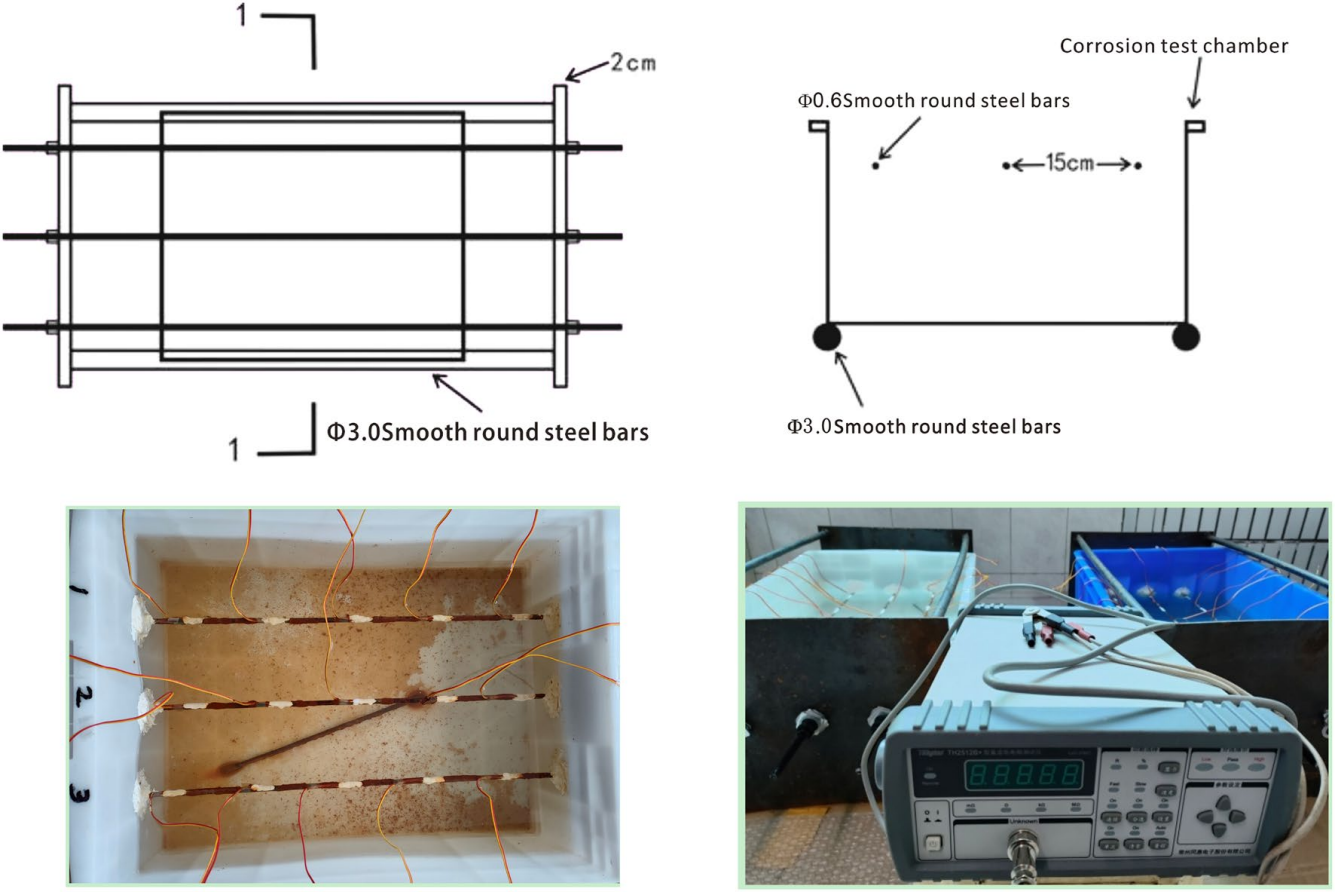
Schematic diagram of corrosion test system and test system.

The test device consists of counterforce frame, test box, nut and anchor bar. Among them, the 500 × 410 × 230 storage box with lid is selected as the test box for holding the corrosion solution; the nut is both a prestressing tensioning device and a locking device for prestressing; the anchor bars are made of 6 mm diameter (Q300) low carbon light source steel as the required strand, and strain gauges are pasted at the anchor bar immersion and the pasted position is treated with water proofing. The test system includes TH2512B+ intelligent DC low resistance tester, PH meter, balance, caliper, tape measure, etc. In which the stress application and the change of anchorage force are observed by a strain gauge connection resistance tester. The corrosion test system and test system are shown in Fig. 1.

### Corrosion anchor bar mechanical properties test

After the corrosion was completed, the anchor bars were de-rusted and dried, and then the mechanical property indexes such as fracture load and elongation of the specimens after corrosion were determined by tensile tests. Tensile tests were performed with reference to the test method (GB/T228.1-2010)^26^.

## Analysis of experimental results

### Corrosion damage occurs, the development of morphological characteristics

By observing the corrosion damage morphology of prestressing anchor tendons at different pH values and different corrosion times, we study the morphological characteristics of corrosion occurrence and development of prestressing anchor tendons at different pH values and different times.

#### Acidic solution

**Figure 2:**
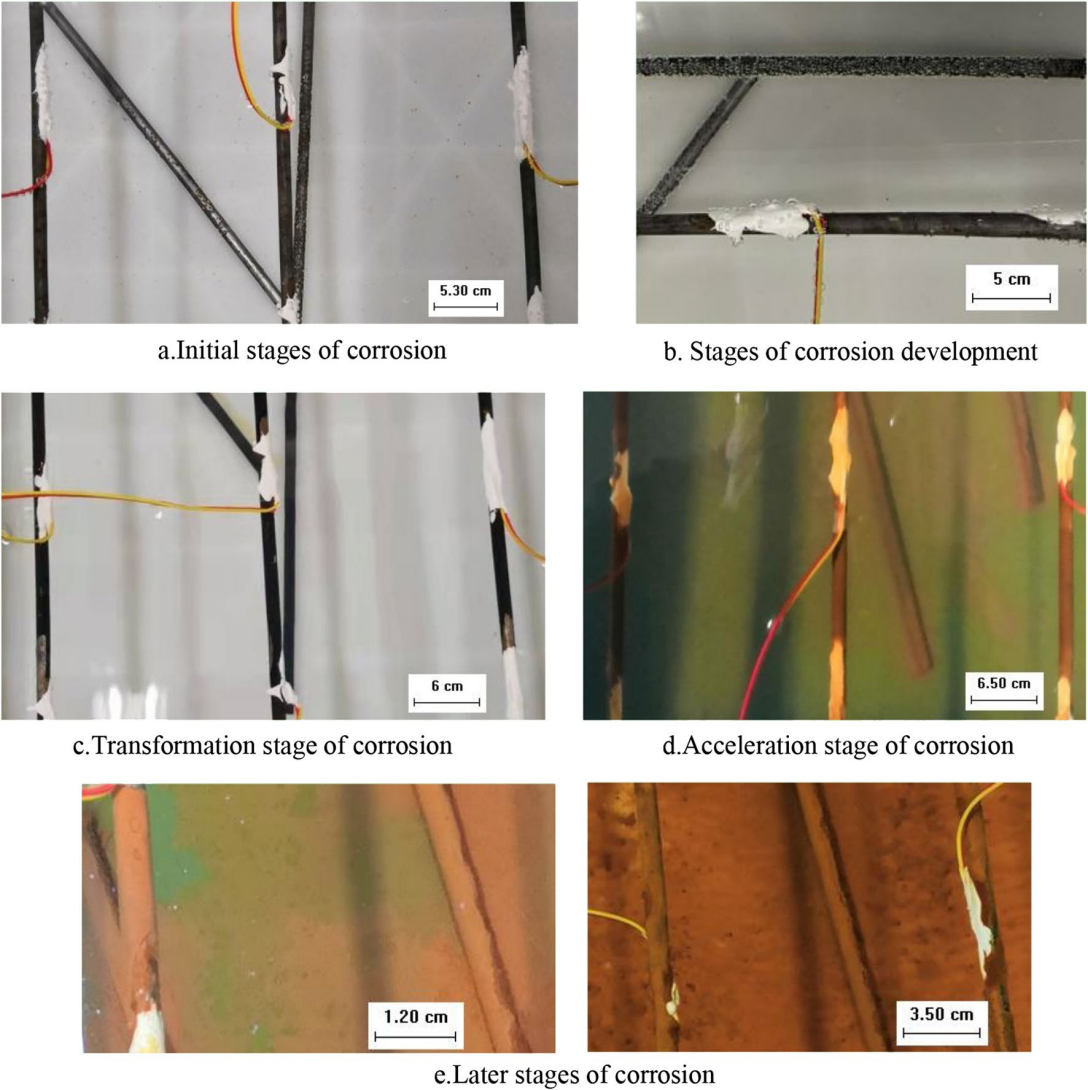
Characteristics of anchor tendon morphology during corrosion occurrence and development (acidic solution).

In the acidic solution with pH 5, the hydrogen precipitation reaction occurs preferentially, and the oxygen absorption reaction starts after the hydrogen precipitation reaction gradually stops (Fig. 2). In the initial stage of corrosion, when the anchor tendon starts to contact with the acidic solution, the H^+^ in the acidic solution reacts with the oxide film preferentially, and a small portion of the anchor tendon matrix reacts with the H^+^ to produce hydrogen gas, which is visible as a layer of small bubbles attached to the anchor tendon surface (Fig. 2a). In the development stage of corrosion, the oxide film on the surface of the anchor tendon gradually fades away and a large number of full-bodied bubbles are attached to its surface (Fig. 2b). During the transition phase of corrosion, the bubbles on the surface of the anchor bars slowly decrease until no bubbles occur. At this time, the anchor bar matrix starts to react with the dissolved O_2_ and H_2_O in the solution by oxygen absorption, and the surface of the anchor bar is gradually covered with a layer of black material (Fig. 2c). The accelerated stage of corrosion, as the corrosion continues to deepen, the anchor tendons occur significant rusting, the surface began to generate a layer of yellow–brown material (Fig. 2d). At the later stage of corrosion, the whole length of the anchor bar was uniformly covered with loose yellow rust, due to the formation of a passivation film, the corrosion rate gradually became slower, and small round hole-like corrosion products were formed in the local area of the anchor bar (Fig. 2e).

#### Neutral solution

**Figure 3:**
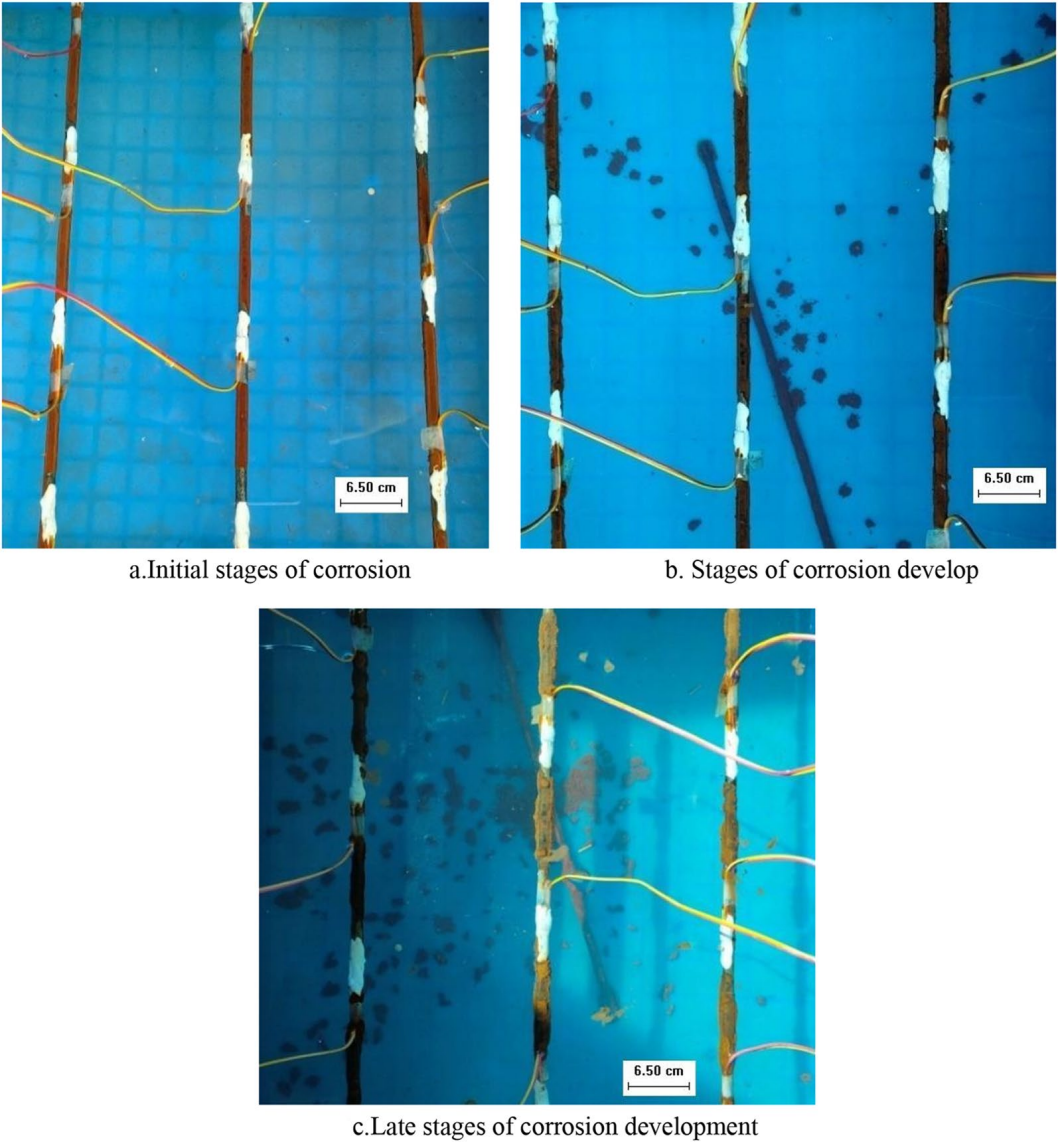
Characteristics of anchor tendon morphology during corrosion occurrence and development (neutral solution).

The prestressed anchor bar in the neutral solution is a simple oxygen absorption reaction (Fig. 3). At the initial stage of corrosion, the rusting was very obvious, and the yellowish-brown rust covered the surface of the anchor reinforcement, which was relatively loose (Fig. 3a). Since the neutral solution in this test contains Cl^−^, which is an extremely strong depassivating agent, it makes the rusting material on the surface of the anchor bar fluffy and loose and facilitates the accelerated corrosion of the anchor bar by O_2_ and H_2_O. Corrosion development stage, with the corrosion of corrosion, the amount of corrosion gradually increased, yellow–brown corrosion substance thickly covered in the surface of the anchor tendons, and corrosion substance distribution is uniform and loose (Fig. 3b). At the later stage of corrosion development, corrosion proceeds slowly, and the surface of the anchor bar is covered with yellow–brown rust and gradually increases and becomes dense (Fig. 3c).

#### Alkaline solution

**Figure 4:**
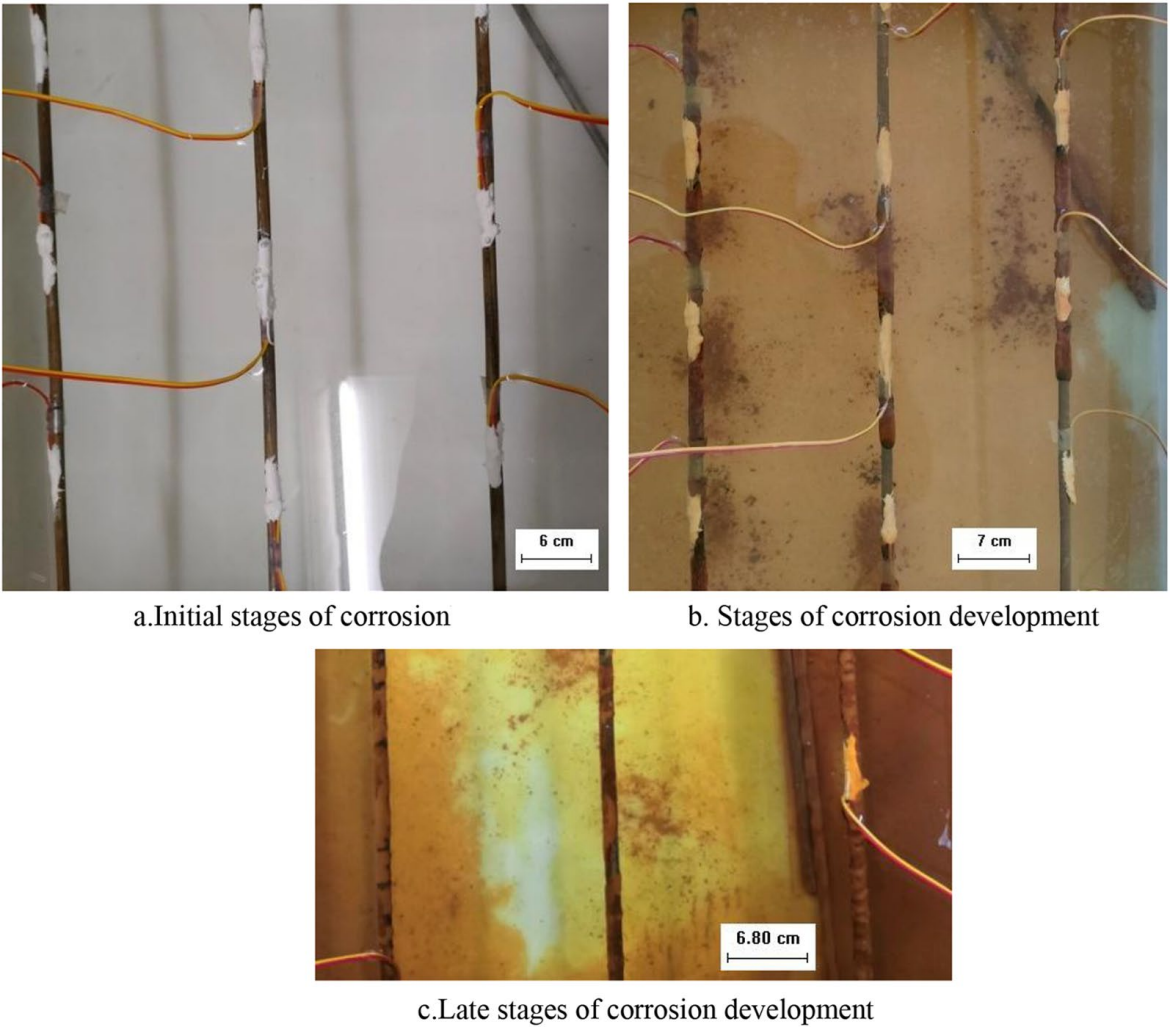
Characteristics of anchor tendon morphology during corrosion occurrence and development (alkaline solution).

In an alkaline solution of pH 9, the prestressing anchor bar underwent an oxygen absorption reaction. However, the alkaline environment acts and the corrosion phenomenon is obviously different from the oxygen absorption corrosion of anchor bars in neutral solution (Fig. 4). In the initial stage of corrosion, the corrosion of anchor bars in alkaline solution is relatively slow, and the anchor bars are basically in a passivated state. At this time, the prestressing anchor bar is slightly corroded in the initial stage, and the surface of the anchor bar still shows a metallic luster (Fig. 4a). Corrosion development stage, as the corrosion reaction proceeds, the anchor bar corrosion material gradually increased, covering the surface of the anchor bar, yellow–brown, uniform and loose distribution (Fig. 4b). Corrosion development of the late stage, the prestressing anchor corrosion slowly, the anchor surface rust continues to increase, thicken, completely covering the anchor surface, rust gradually dense, yellow–brown (Fig. 4c).

### Analysis of the relationship between unit length corrosion weight and various factors

By measuring the corrosion weight per unit length of anchor bars, Tables 3 and 4 show the corrosion weight per unit length of anchor ropes for 3 and 6 months under different stress levels and pH values, respectively. According to the change of corrosion weight per unit length of anchor bars under the action of different influencing factors, the change law of corrosion weight per unit length with stress level, pH value and time was studied respectively.

**Table 3:** Corrosion weight per unit length of different anchor bars for 3 months of corrosion.

pH	Corrosion weight per unit length (g cm^−1^)							
	No prestressing		24% stress level		36% stress level		48% stress level	
	Measured value	Average value	Measured value	Average value	Measured value	Average value	Measured value	Average value
5.0	0.057	0.055	0.074	0.071	0.075	0.078	0.092	0.097
	0.060		0.076		0.086		0.110	
	0.048		0.063		0.073		0.089	
7.0	0.036	0.031	0.040	0.033	0.050	0.043	0.056	0.047
	0.033		0.032		0.040		0.044	
	0.024		0.027		0.039		0.041	
9.0	0.026	0.016	0.028	0.019	0.026	0.020	0.026	0.019
	0.012		0.016		0.018		0.016	
	0.010		0.013		0.016		0.015

**Table 4:** Corrosion weight per unit length of different anchor bars for 6 months of corrosion.

pH	Corrosion weight per unit length (g cm^−1^)							
	No prestressing		24% stress level		36% stress level		48% stress level	
	Measured value	Average value	Measured value	Average value	Measured value	Average value	Measured value	Average value
5.0	0.103	0.093	0.116	0.108	0.118	0.109	0.110	0.102
	0.087		0.108		0.110		0.100	
	0.089		0.100		0.099		0.096	
7.0	0.053	0.051	0.083	0.072	0.088	0.078	0.085	0.076
	0.052		0.068		0.074		0.072	
	0.048		0.065		0.072		0.071	
9.0	0.028	0.02	0.057	0.053	0.059	0.056	0.056	0.050
	0.017		0.053		0.056		0.050	
	0.015		0.049		0.053		0.044	

#### The relationship between corrosion weight per unit length and the level of stress

**Figure 5:**
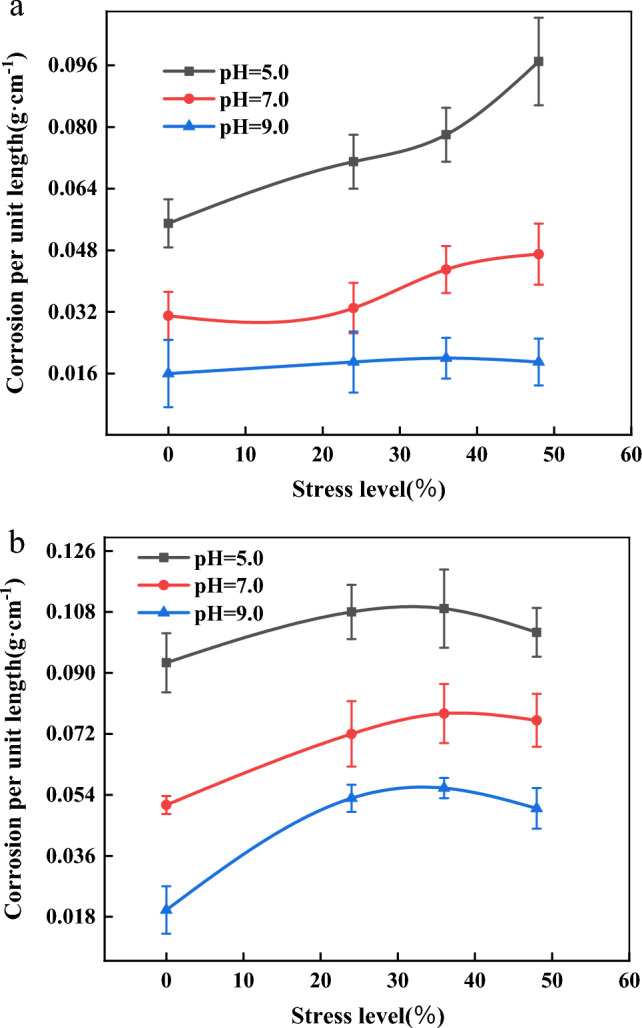
Relation curve between corrosion weight per unit length and stress level. (**a**) Relation curve between corrosion weight per unit length and stress of different anchor bars corroded for 3 months. (**b**) Relation curve between corrosion weight per unit length and stress of different anchor bars corroded for 6 months.

Figure 5 show the curves of corrosion weight per unit length versus stress level when pH is 5, 7 and 9. As seen in Tables 3, 4 and Fig. 5 (the values in the curve consist of the mean and standard deviation), the dispersion of the corrosion weight per unit length is small. As can be seen in Fig. 5a, the corrosion solution is acidic (at pH 5), and the corrosion weight per unit length of anchor bars shows an increasing trend as the stress level increases. The weight of corrosion per unit length at 48% stress level is 1.8 times more than the weight of corrosion under unstressed conditions. The solution was neutral and alkaline (at pH 7 and 9), and as the stress level increased, the corrosion weight per unit length of anchor tendons tended to increase slowly but not significantly. From Fig. 5b can be seen, with the increase in stress level, the unit length of corrosion weight is increasing, but its corrosion increment is gradually decreasing, when the stress level increases to a certain extent (36%), the unit length of corrosion weight are showing a decreasing trend. This is due to the anchor tendons in the corrosion process, stress relaxation occurs, for the corrosion products have been produced, a certain compression deformation will occur, making its pore structure affected, become dense, thereby reducing the corrosion rate.

#### The relationship between corrosion weight per unit length and corrosion pH

**Figure 6:**
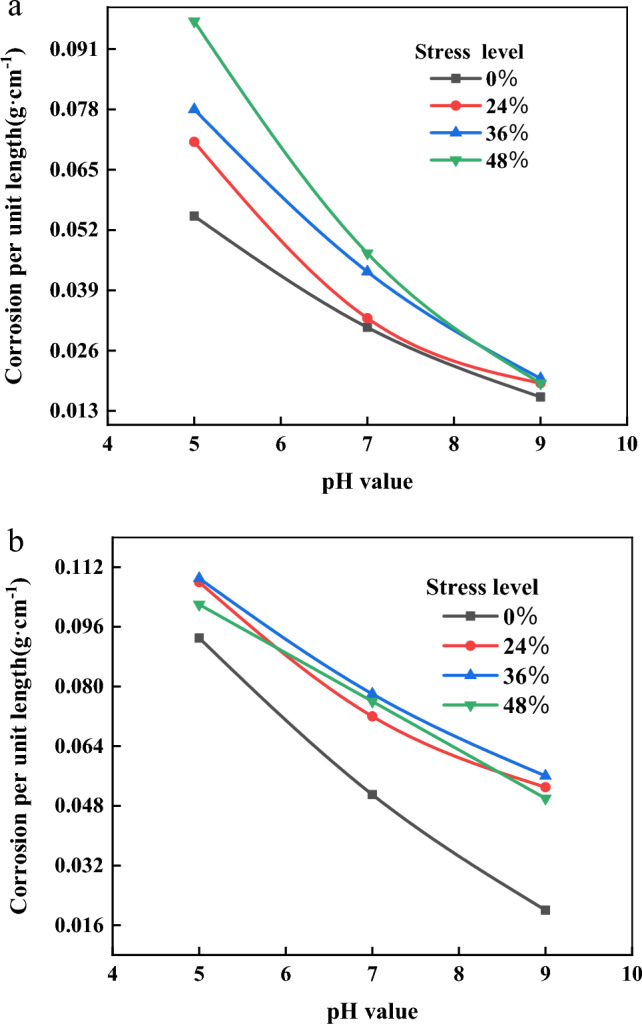
Relation curve between corrosion weight per unit length and pH. (**a**) Relation curve between corrosion weight per unit length of different anchor bars corroded for 3 months and corrosion pH value. (**b**) Relation curve between corrosion weight per unit length of different anchor bars corroded for 6 months and corrosion pH value.

Figure 6 show the corrosion weight per unit length versus pH curves for anchor bars immersed in corrosion solution for 3 and 6 months, respectively. It can be seen from the figure that when the pH value is 5, 7 and 9, the corrosion weight per unit length of anchor tendon has the same trend, that is, with the increase of pH value, the corrosion weight per unit length of anchor tendon is decreasing, and the overall trend is in the form of negative exponential. The weight of corrosion per unit length at pH 5 is 2–5 times higher than that at pH 9. Therefore, especially for small diameter anchor bars, a smaller change in pH will result in a significant change in their final life. In actual engineering, with the gradual alkalization of the corrosive environment, the final service life of the anchorage project will increase geometrically. The main reason for this phenomenon is that the corrosion process is constantly generating corrosion products, attached to the surface of the anchor bar, forming a passivation film, and in an alkaline environment is more likely to form Fe_2_O_3_ or Fe_3_O_4_ passivation film, passivation film can inhibit the further development of corrosion to the interior of the anchor bar.

#### The relationship between the weight of corrosion per unit length and pH and time coupling

**Figure 7:**
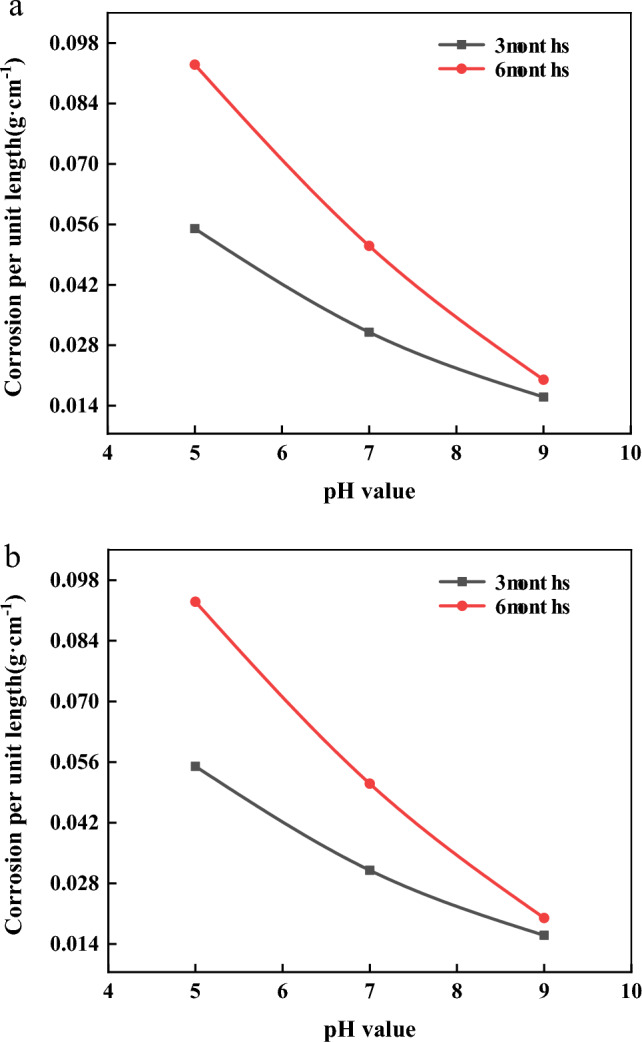
Relation curves between the corrosion amount per unit length of anchor bar and pH value in unstressed state and prestressed state, respectively. (**a**) Relation curve between corrosion weight per unit length and time under unstressed state. (**b**) Curve of corrosion weight per unit length versus time under prestressed condition (48 stress level)

Figure 7 shows the curves of corrosion weight per unit length of anchor bars versus pH for the unstressed and prestressed conditions (48% stress level). It can be seen from the figure that the corrosion weight per unit length shows a decreasing trend with the increase of pH. Under the influence of different pH values, the corrosion of anchor tendons in acidic solution is the largest, followed by that in neutral solution and the smallest in alkaline solution. With the growth of time, the weight of corrosion per unit length will continue to increase, but the increment of corrosion gradually decreases, this trend of corrosion increment in the alkaline solution is more significant, indicating that the alkaline solution than the acidic solution is easier to form a passivation film on the surface of the anchor bar. The effect of pH and time on anchor bar corrosion was significant, i.e. the effect of pH and time coupling was stronger for the weight of corrosion per unit length of anchor bar.

### Analysis of the relationship between anchorage force and corrosion time

**Figure 8:**
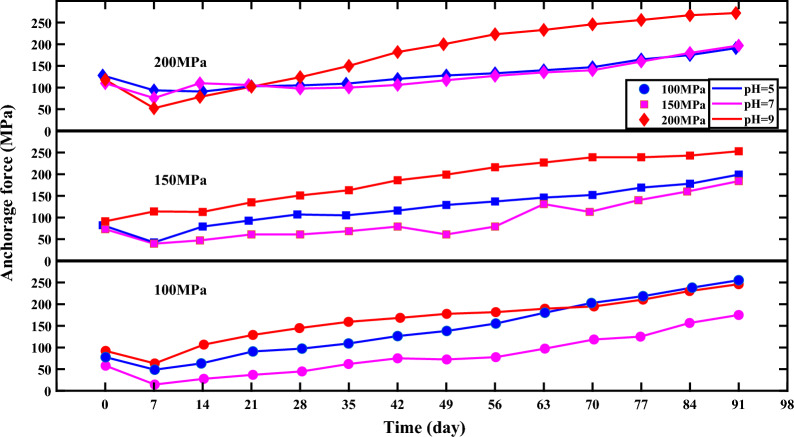
Curve of anchorage force with time under different corrosive environments.

The change in prestress was monitored by a strain gauge connection resistance tester, and the change in anchorage force with time after 3 months of study corrosion is shown in Table 5 with Fig. 8.

**Table 5:** Changes in anchorage force under different corrosive environments.

Time (days)	Change in anchorage force								
	24% stress level (100 MPa)			36% stress level (150 MPa)			48% stress level (200 MPa)		
	pH 5	pH 7	pH 9	pH 5	pH 7	pH 9	pH 5	pH 7	pH 9
0	78	58	92	82	73	91	128	110	118
7	49	14	63	43	40	114	94	76	52
14	63	28	107	79	47	113	91	110	78
21	91	37	129	93	61	135	103	106	102
28	97	45	145	107	61	151	105	98	124
35	109	62	159	105	68	163	109	100	150
42	126	75	168	116	79	186	120	106	182
49	138	72	178	129	61	199	128	117	120
56	155	78	182	137	79	216	133	127	223
63	180	97	189	146	131	227	140	135	233
70	203	118	195	152	113	239	147	140	246
77	218	125	211	169	140	239	165	160	256
84	238	157	230	178	160	243	175	180	267
91	255	175	246	199	184	253	191	197	272

Figure 8 shows the curve of anchor force change process during the corrosion of anchor bars. From the figure, it can be seen that the change in anchorage force during the corrosion of anchor bars shows the following characteristics.

1. With the increase of corrosion time, the anchor reinforcement anchorage force changes mainly through 2 stages of rapid decline and slow rise. The rapid descent stage is mainly affected by the adjustment of steel plate force deformation, anchor bar relaxation and the frictional resistance between the nut and anchor bar, resulting in a large loss of anchorage force. After the above process is completed, the anchorage force no longer decreases, mainly because the steel plate adapts to deformation and corrosion under long-term pressure causing damage to the cross-sectional area of the anchor tendons, resulting in a rise in pre-stress.
2. At 24% stress level (100 MPa), the anchor tendon anchorage force rises fastest in the acidic solution, followed by the alkaline solution and slowest in the neutral solution. At 36% stress level (150 MPa), the anchor tendon anchorage force rises fastest in the alkaline solution, followed by the acidic solution and slowest in the neutral solution. At 48% stress level (200 MPa), the anchor tendon anchorage force rises fastest in the alkaline solution, followed by the neutral solution and slowest in the acidic solution.

### Analysis of the relationship between the mechanical properties of corroded anchor bars and various factors

By testing 2 mechanical property indexes, fracture load and elongation, on corroded anchor bars, the changes of fracture load and elongation with stress level and pH value were studied, respectively.

Tensile tests were performed on the anchor bars before and after corrosion. The mechanical properties of the anchor bars before corrosion are shown in Table 6. The fracture loads of the anchor bars after corrosion under different conditions are shown in Table 7. The elongation of anchor bars after corrosion under different conditions is shown in Table 8.

**Table 6:** Mechanical property parameters of anchor bars before corrosion.

Elongation after break (%)	Fracture load (kN)
25	11.9

**Table 7:** Fracture load of anchor bars after corrosion under different conditions.

pH	Fracture load (kN)							
	No prestressing		24% stress level		36% stress level		48% stress level	
	Measured value	Average value	Measured value	Average value	Measured value	Average value	Measured value	Average value
5.0	11.7	11.5	11.0	10.7	10.8	10.8	10.7	10.5
	11.5		10.6		11.2		10.6	
	11.3		10.5		10.4		10.2	
7.0	12.2	11.9	12.0	11.9	12.5	12	12.2	11.9
	11.8		11.8		11.7		11.6	
	11.7		11.9		11.8		11.9	
9.0	11.9	11.8	11.8	11.7	12.1	11.7	11.7	11.4
	11.8		11.7		11.6		11.2	
	11.7		11.6		11.4		11.3

**Table 8:** Elongation of anchor bars after corrosion under different conditions.

pH	Elongation (%)							
	No prestressing		24% stress level		36% stress level		48% stress level	
	Measured value	Average value	Measured value	Average value	Measured value	Average value	Measured value	Average value
5.0	21.0	20.0	17.6	17.0	16.9	16.0	16.0	15.0
	19.6		16.9		15.6		14.7	
	19.4		16.5		15.5		14.3	
7.0	25.8	25.0	23.7	23.0	25.6	25.0	25.5	25.0
	24.7		22.8		24.6		24.5	
	24.5		22.5		24.8		25.0	
9.0	24.5	24.0	25.0	24.0	23.8	23.0	24.6	24.0
	24.4		23.7		22.7		24.1	
	23.1		23.3		22.5		23.3	

#### Analysis of the relationship between the corrosion anchor bar fracture load and each factor

1. The relationship between fracture load and corrosion stress level

**Figure 9:**
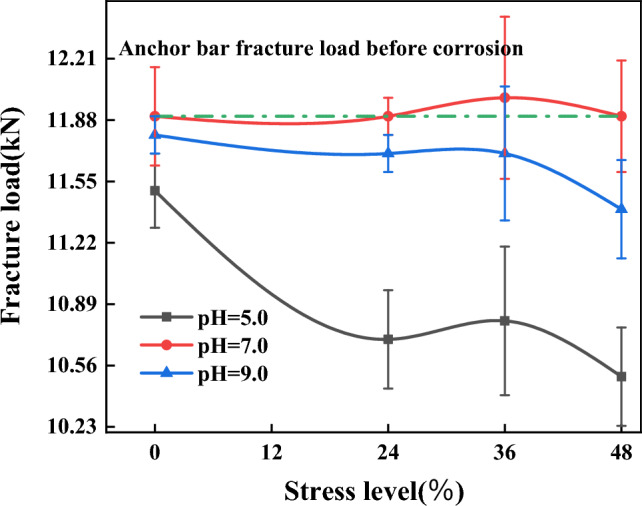
Relation curve between fracture load and stress level of corroded anchor bars.

Figure 9 shows the relationship between the fracture load and the stress level of the anchor bar after 3 months of corrosion. As shown in Table 7 and Fig. 9 (the values in the curves consist of the mean and standard deviation), the dispersion of the corrosion anchor bar fracture load is low. From the figure, it can be seen that the fracture loads of anchor bars at different stress levels exhibits the following characteristics.

1. When the corrosion solution is acidic (pH 5), the anchor bar fracture load decreases more significantly as the stress level increases. After corrosion, the fracture load of anchor bars with prestressing applied was significantly smaller than that of anchor bars without prestressing, and the reduction of fracture load was significantly larger than that of anchor bars without stressing. As shown in Table 7, the fracture load reduction without prestressing anchor bar at pH 5 is 0.4 kN, while the fracture load reduction with prestressing anchor bar applied is more than 1 kN, which is more than twice of the fracture load reduction without prestressing anchor bar. This is due to the micro-cracking of the surface of the anchor reinforcement under tensile stress, the surface of the damaged anchor reinforcement and the undamaged surface form the anodic area and cathodic area respectively, constituting a corrosion cell, the metal in the anodic area dissolves and generates current flow to the cathode. And the anode area is small, the cathode area is large, resulting in a large current density in the anode area, which further corrodes the damaged surface and gradually forms pitting pits, which are stress concentration points, thus gradually forming cracks at the damage, thus causing the corrosion of the prestressed anchor reinforcement to be more serious than that of the non-prestressed anchor reinforcement.
2. The reduction of anchor bar fracture load with stress level after corrosion did not show a clear monotonic characteristic when the corrosion solution was neutral (pH 7) and when the corrosion solution was alkaline (pH 9) (Fig. 9). This is due to the pH value of 7, different stress levels under the anchor bar corrosion occurs very quickly, yellow–brown corrosion quickly covered the surface of the anchor bar, the formation of passivation film, resulting in a small weight of corrosion of the anchor bar, pH value of 9, the anchor bar corrosion phenomenon is relatively slow, the initial stage is basically in the passivation state, the anchor bar corrosion weight is small, the corrosion weight is small resulting in the anchor bar fracture load is not obvious.
3. Relationship between fracture load and corrosion pH

**Figure 10:**
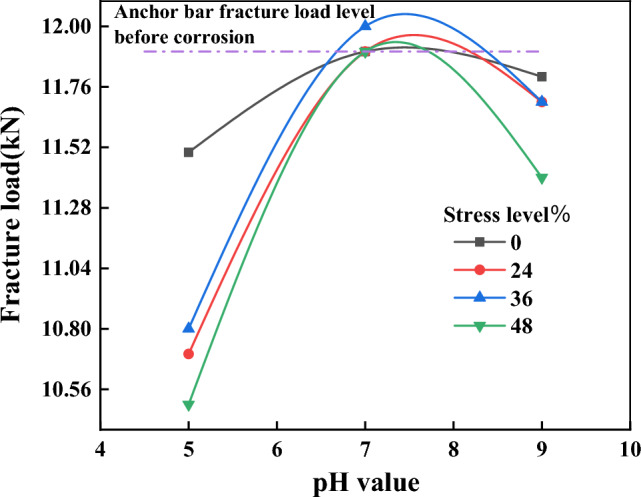
Relation curve between fracture load of corroded anchor bars and corrosion pH value.

Figure 10 shows the relationship between the fracture load of anchor bars and corrosion pH after 3 months of corrosion. From the figure, it can be seen that the smaller the pH of the corrosion solution, the more obvious the reduction of the fracture load of the anchor bar after corrosion. The fracture load of anchor tendons after corrosion with acidic solution of pH 5.0 showed a significant reduction, the fracture load of anchor tendons after corrosion with neutral solution of pH 7.0 showed no significant change compared with that before corrosion, and the fracture load of anchor tendons after corrosion with alkaline solution of pH 9.0 showed a certain degree of reduction. As shown in Table 7, when the pH value was 5.0 and the stress level was 48%, the fracture load of the anchor bar after corrosion was the smallest, which was 10.5 KN, which was 12% lower than that before corrosion.

#### Analysis of the relationship between elongation and various factors

**Figure 11:**
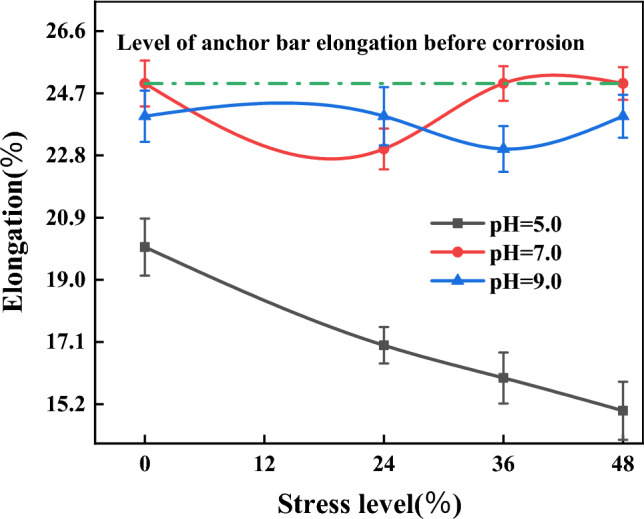
Relationship curve of corrosion anchor bar elongation and stress level.

1. Relationship between elongation and corrosion stress level
  Figure 11 shows the curve of anchor bar elongation versus stress level after 3 months of corrosion. From Table 8 and Fig. 11 (the values in the curves consist of the mean and standard deviation), it can be seen that the degree of dispersion of the elongation of the anchor bars after corrosion is not significant. From the figure, it can be seen that the elongation of anchor bars under different stress levels exhibits the following characteristics.
  1. When the corrosion solution is acidic (pH 5), the loss of anchor bar elongation becomes more pronounced as the stress level increases. The amount of elongation loss after corrosion of anchor bars with prestress applied is greater than that of anchor bars without stress, and the amount of elongation loss after corrosion of anchor bars with large prestress is greater than that of bars with small stress (Table 8). This is due to the coupling effect of electrochemical corrosion and stress, the anchor tendons show cross-sectional damage and stress concentration, resulting in a reduction of their mechanical properties and loss of elongation.
  2. When the corrosion solution is neutral (pH 7), the relationship curve between anchor bar elongation and stress level is approximately horizontal straight line, and there is basically no loss of anchor bar elongation after corrosion by immersion in neutral solution (Table 8), indicating that the weight of corrosion of prestressing anchor bars in neutral solution is small. This is because the degree of corrosion damage depends not only on the magnitude of the stress level, but also with the rate of oxygen absorption corrosion. Due to the initial stage of corrosion, the rust is very obvious and the yellowish-brown rust covers the surface of the anchor bar, which hinders the corrosion to a certain extent and leads to a small amount of corrosion of the anchor bar. At 36% and 48% stress levels, the corrosion rate is even faster, and the rate of rust build-up exceeds even its rate of breaking the passivation film. Therefore, the elongation of anchor bars after corrosion at 36% and 48% stress levels is greater than that of anchor bars at 24% stress level.
  3. When the corrosion solution was alkaline (pH 9), the anchor bar elongation and stress level showed fluctuating changes and did not exhibit monotonic characteristics. In more agreement with the previously obtained law, the loss of anchor bar elongation after soaking in alkaline solution was not significant.
2. Relationship between elongation and corrosion pH

From Tables 6 and 8 can be seen that after corrosion under acidic conditions, there is a significant loss of anchor bar elongation, the anchor bar elongation before corrosion is 25%, and after corrosion in acidic solution its elongation are less than 25%, after corrosion by immersion in neutral solution the anchor bar elongation damage is not obvious, and after corrosion in alkaline solution its elongation damage is smaller.

**Figure 12:**
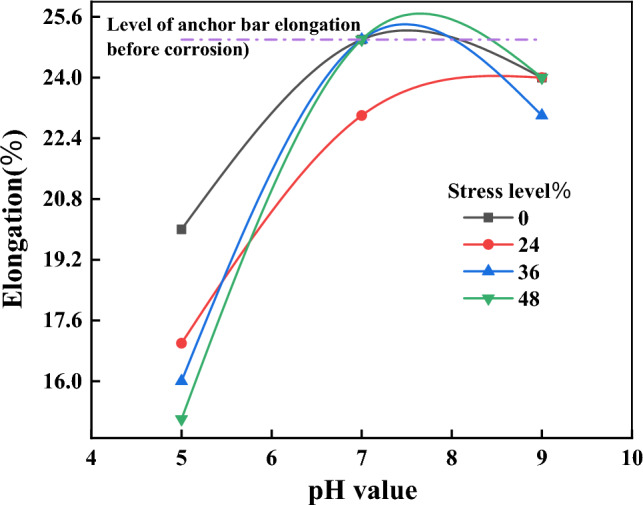
The relation curve of corrosion anchor bar elongation and corrosion pH value.

Figure 12 shows the relationship curve between the elongation of anchor tendons and corrosion pH after 3 months of corrosion. As can be seen from the figure, the smaller the pH of the corrosion solution, the more significant the loss of elongation of the anchor bars after corrosion. The anchor bar elongation after corrosion by immersion in acidic solution at pH 5 showed significant loss, the anchor bar elongation after corrosion by immersion in neutral solution at pH 7 showed no significant damage compared with that before corrosion, and the anchor bar elongation after corrosion by immersion in neutral solution at pH 9 showed some degree of damage; among them, the anchor bar elongation after corrosion by immersion at pH 5 and stress level of 48% showed the greatest damage, with a 40% decrease compared with the elongation before corrosion.

## Conclusion and outlook

Based on indoor accelerated corrosion tests of prestressing anchor bars and tensile tests of corroded specimens, the evolution of the performance of prestressing anchor cables under the action of corrosive media was studied and the following conclusions were obtained.

1. The initial stage of corrosion, prestressing anchor bars in neutral solution corrosion rust phenomenon is very obvious, acidic solution is the second, alkaline solution is basically in the passivation state. With the continuation of corrosion, the acidic solution corrosion damage to the anchor tendon is obvious, and the corrosion of the anchor tendon is not obvious after the alkaline solution immersion.
2. The longer the corrosion time, the more serious the degree of corrosion damage to the anchor bars. The external characteristics of the occurrence and development of corrosion of anchor tendons are the gradual loss of luster, yellow–brown rust covering the surface of anchor tendons, etc. The mechanical property damage is reflected in the reduction of fracture load, elongation and changes in anchorage force.
3. In the three corrosive media, the weight of corrosion per unit length increases as the pH value becomes smaller, reflecting the corrosion damage as uniform corrosion; while the fracture load and post-fracture elongation of alkaline solution is less than that of neutral solution, which is due to the stress concentration caused by local corrosion of anchor tendons in alkaline solution, resulting in the reduction of fracture load and post-fracture elongation.
4. In the three corrosive media, the higher the stress level of the anchor bar, the more serious the corrosion degree of the anchor bar, especially in the acid solution, the corrosion weight per unit length of the anchor bar at the stress level of 48% is 1.1 times of the corrosion weight under the condition of no stress, the fracture load reduction and elongation loss rate are two times of the condition of no stress.

In addition to its own high stress on the corrosion of the prestressed anchorage structure, the corrosive environment in which it is endowed is quite different from that of the prestressed concrete structure, including the erosive media in the geotechnical medium and groundwater in the anchorage structure environment, the bimetallic effect and stray currents in the strata. Therefore, the use of suitable accelerated corrosion test methods to explore the corrosion pattern and failure mechanism of prestressed anchorage structures under the joint action of environmental corrosion factors and mechanics is a problem worth studying. However, the test conditions in this paper cannot yet fully simulate the actual geotechnical engineering environment, such as groundwater seepage, oxygen, and pH in the geotechnical engineering environment can cause corrosion to the anchorage engineering structure. In addition, local corrosion of the prestressing anchor bars can aggravate the corrosion of the anchored structure. Therefore, it is the direction of further research on the service performance of prestressing anchorage structures to consider the coupling of physical, chemical and seepage environments to carry out experimental research on corrosion damage of prestressing anchor ropes (rods) and to carry out research on the local corrosion behavior and characteristics of prestressing anchor ropes (rods) containing defects. In addition, on the accelerated corrosion test of prestressing anchorage structure, mostly using immersion corrosion, the test period is long, and easily affected by the experimental conditions. How to study the long-term evolution of anchor tendon performance decay in anchorage system by electrochemical corrosion test, shorten the test period and enhance the controllability of the test is an important problem to be solved in the study of durability of prestressed anchorage structure.

## Data Availability

The datasets analysed during the current study are available from the corresponding author on reasonable request.
